# Environmental Health Knowledge Does Not Necessarily Translate to Action in Youth

**DOI:** 10.3390/ijerph20053971

**Published:** 2023-02-23

**Authors:** Shereen Elshaer, Lisa J. Martin, Theresa A. Baker, Erin Roberts, Paola Rios-Santiago, Ross Kaufhold, Melinda Butsch Kovacic

**Affiliations:** 1 Cincinnati Children’s Hospital Medical Center, Department of Pediatrics, University of Cincinnati College of Medicine, Cincinnati, OH 45229, USA; 2 Department of Public Health and Preventive Medicine, Mansoura University Faculty of Medicine, Mansoura City 35516, Egypt; 3 Shriver National Institute of Child Health and Human Development, Bethesda, MD 20847, USA; 4 Department of Rehabilitation, Exercise, and Nutrition Sciences, The University of Cincinnati College of Allied Health Sciences, Cincinnati, OH 45267, USA

**Keywords:** local environment, knowledge, environmental health, behavior, children

## Abstract

Environmental challenges pose serious health problems, especially for children, and lay public action is lacking. This study sought to characterize the relationship between environmental health knowledge and behavior in youth. A cross-sectional, descriptive survey with quantitative and qualitative questions was conducted. Open-ended questions were coded to generate themes/subthemes. Subscales’ scores were presented as mean ± SD or median and interquartile range (IQR). T- and Mann–Whitney tests were used to compare groups, and correlations were used to evaluate covariation. A total of 452 children were surveyed. Youth verbalized concerns about their environments and their impact on health. Air pollution was the most concerning issue. Participants had moderate knowledge scores. Few described the three health domains; even fewer included environment. Behavior scores were low and weakly correlated with knowledge, but were moderately correlated with attitude and self-efficacy. Participation in environmental classes, activities, and clubs was associated with higher scores. We found variable environmental health knowledge, limited understanding of the local environment’s impact on health, and a weak association between youth’s knowledge and behavior. Focused formal and non-formal educational experiences were associated with improved scores, indicating the value of targeted youth educational programming to increase environmental health knowledge and action.

## 1. Introduction

Over 12 million people worldwide die because they live or work in unhealthy environments, and many more experience morbidities attributable to modifiable environmental conditions [1]. Notably, youth have greater vulnerability to adverse health outcomes linked to neighborhood and environmental health hazards [2,3]. Specifically, environmental pollutants have been linked to asthma [4,5], ADHD [6,7], and poor cognitive functioning in youth [8,9]. Economic costs for these preventable conditions exceed 50 billion dollars annually for American children alone [10]. Thus, it is not surprising that the US government has environmental health goals in the Healthy People 2030 objectives, which focuses on reducing people’s exposure to harmful pollutants in the air, water, soil, food, and materials in homes and workplaces [11].

While experts have a clear consensus that environmental health is a serious problem in the US, recognizing environmental health issues by the lay public is critical to move from research to action [12,13]. In the past decade, there has been an increased focus on the generation of environmental health content for primary (K-12) education, yet it is rarely paired with human health education in schools (aka environmental health) [14,15]. Additionally, while health is considered a priority area, student comprehension is not tested; thus, implementing health and environmental health programming can be valuable [5,14,15]. Youth often rely on television, books, newspapers, and friends as primary environmental information sources [16].

Furthermore, environmental health concerns are infrequently paired with concrete ways to apply the knowledge learned to identify possible solutions, especially when discussing local challenges [17]. In a study of pregnant women, many participants recognized environmental health concerns; however, many participants felt powerless in changing outcomes [18]. The sense of powerlessness was more common in younger participants. This may be why studies have shown that the media and news are ineffective in empowering and engaging youth [19,20].

School-aged youths’ attitudes and concerns about the environment have both direct and indirect influences on their future decision-making and willingness to advocate [21,22]; therefore, understanding the factors that drive their behaviors is critical. Some studies have argued that attitudes rather than knowledge predict behavior in youth. However, the relationship between knowledge, attitude, and behavior is complex [23] and likely varies by age [11]. The complexity of this relationship necessitates further studies.

This study sought to characterize the relationship between environmental health knowledge and behavior in youth. To accomplish this objective, we surveyed youths aged 9–18 years old and examined changes in their responses across three distinct age groups to both quantitative and qualitative questions regarding knowledge, behaviors, attitudes, and self-efficacy of health and environmental health. We designed our study to leverage students from well-resourced schools to maximize the possibility of having students with higher environmental health knowledge because socioeconomic status is positively associated with environmental health knowledge [24,25,26]. These schools are also more likely to have unique offerings and non-formal opportunities focused on environmental health.

## 2. Materials and Methods

### 2.1. Study Design

A cross-sectional, descriptive survey study with both quantitative and qualitative questions was conducted on a convenience sample of Midwestern children and adolescents aged 9 to 18 years old. The Cincinnati Children’s Hospital Medical Center (CCHMC) Institutional Review Board reviewed and approved the study. Because schools are a convenient source of youth, youth within three willing schools representing the east, west, and southern regions of the Cincinnati, Ohio Metropolitan area were invited to participate in this study. Youth from one private school, which taught kindergarten through grade 8, were included, though only children in grades 4 to 8 were queried. Two high schools (one public and one private that only enrolls females) included adolescents in grades 9 through 12. All were adequately resourced, successful schools, and both high schools offered formal and non-formal environmental program options. None of the participating schools nor the Greater Cincinnati area had recently experienced environmental challenges at the time of the study (2015–2016).

Six participating science teachers consented to the study and completed a teacher survey. Each was asked to introduce and administer the survey to their students after sending an informational letter home to parents/guardians one week before the survey was administered. The letter explained the purpose and requirements of the study. Parents could opt their children out of participation by contacting their children’s teachers. However, no parent opted their child out. Assent language was included along with the survey and read aloud by the teacher before the survey was administered. Participating teachers then offered to answer any questions about the purpose and requirements of participating in the study. Following the administration of the survey and its analysis, each participating teacher received a standard report that included a summary of their respective students’ performance on the different subscales. The report’s purpose was to guide the teachers in better tailoring future curricula for their students on the topic of environmental health if they wished to. Providing the report was a benefit to the teachers, but was not a study outcome.

Youth participants indicated their assent to participate in this study by completing the survey. No signed documentation was required from parents/guardians since no identifiable information was collected. The middle school participants (ages 12 to 14) were surveyed first via a paper survey during their science course. All other participants took the survey on school-provided computers or mobile devices (iPads) at a time chosen by their teachers. It took an average of 15 min for participants to complete the survey. In total, 503 participants provided survey responses. Those with missing subscale responses were excluded (47 surveys total, mostly from paper surveys). Four surveys were further excluded from the analysis due to missing information on participants’ ages. Quantitative analyses were performed on 438 surveys; 14 were excluded due to additional incomplete data, while qualitative analysis was performed on 452 surveys.

### 2.2. Surveys

The initial participant survey utilized in this study was modeled off the study by Naquin et al., which examined the environmental knowledge, attitudes, and behaviors of school children in grades 4 through 8 in Louisiana [27]. The survey questions were initially developed for children and adolescents aged 9 to 14 years old (children grades 4 through 8). Similar to ours, Naquin’s questions were both open-ended and discrete. Because the Naquin survey primarily focused on the environment rather than environmental health, some questions were adapted, and new questions about the effects of the environment on health were added. Examples were also included in many of the questions; both positively worded and negatively worded statements were included. A group of researchers (an environmental health professional, an epidemiologist, a public health student, and a toxicologist) reviewed and revised the survey before use. Demographic questions were also included, including a question asking participants if none, one, or both parents worked outside the home (had a job). This was incorporated as a proxy (though imperfect) for economic status, given we were querying children from diverse economic backgrounds. Asthma status was also captured, given children with asthma are often known to receive education related to environmental triggers.

The survey that was given to 9 to 14-year-old youth assessed knowledge (15 questions), attitudes (12 questions), and behaviors (12 questions) towards the environment and health in a personal context, as well as their perceptions of their community’s health, and how strongly their behaviors impacted their environment. Knowledge questions covered pollution types and their effects on health, pollution sources, and pro-environmental behaviors. In attitude questions, respondents were asked about their views of personal healthy and pro-environmental behaviors and of their immediate community (family, friends, and school teacher) toward their environment. Behavioral questions examined respondents’ littering, recycling, energy conservation, and healthy habits such as eating, exercising, and personal hygiene. Thereafter, the survey was slightly modified, and the self-efficacy subscale was added “given its importance in human motivation and behavior” [28,29]. The modified survey was given to 15 to 18-year-olds and included: knowledge (16 questions), attitude (12 questions), behavior (12 questions), and self-efficacy (12 questions) subscales. The self-efficacy subscale contained questions about the youth’s beliefs about their ability to be involved in healthy and environmentally friendly behaviors.

Responses were recorded on a 5-point Likert-type scale. Participants could “Strongly agree,” “Agree,” “Neither agree nor disagree,” “Disagree,” or “Strongly disagree” to each question. Values of 2, 1, 0, −1, and −2 were assigned to the responses, respectively, and a higher numerical score reflected a more positive response. The coding of the items containing negative statements was reversed in the analysis process, i.e., 2 (strongly agree) to −2 (strongly disagree). Higher scores were favorable; percentages were calculated based on the total number of possible points per subscale. A section of the survey following the subscales queried the participants’ sex, age, grade, residence, parents’ employment status, and taking any classes and/or participating in clubs or activities specifically on the topic of environmental science or environmental health.

The survey ended with four open-ended questions seeking to explore the participants’ understanding and perceptions of the term health, what worried them most about the environment, and what they could do to help the environment. We asked these questions after the Likert scale questions, which offered participants various ways human health could be affected by the environment. After responding to our Likert scale questions, we wanted to see if the students could bridge health to the environment and understand and use this information to respond to our qualitative questions. We considered that they might better understand health and its connection with the environment after answering the Likert scale questions. Feedback questions about the survey were included.

The total scale internal consistency for the participant survey was based on Cronbach’s alpha for the initial survey given to participants aged 9 to 14 years old and the survey for older participants aged 15 to 18 years old were 0.87 and 0.89, respectively. For the initial survey, the internal consistency for individual knowledge, attitude, and behavior subscales were 0.79, 0.80, and 0.76, respectively, which indicated that the subscales are reliable. For the survey containing self-efficacy (ages 15 to 18 years old), the internal consistency of the individual knowledge, self-efficacy, attitude, and behavior subscales was 0.79, 0.87, 0.76, and 0.62, respectively, which indicated that the subscales are reliable.

The participating teachers were also asked to complete a six-question survey. We asked teachers about the grade level and subject(s)/topic(s) they taught. They were also asked about the main environmental topics they teach and if they specifically discuss with their students how the environment affects their health, and if so, what approaches they used. Out of the six teachers, only one indicated that they had integrated how the environment affects health into their teaching. One teacher also mentioned that they had not directly linked the environment to health in their classes based on the assumption that students make the connection independently. Finally, all participating teachers indicated that they had previously taught their students about environment-related topics such as air pollution, solid waste, wastewater, and recycling.

### 2.3. Analysis

Survey responses were collected and managed using REDCap electronic data capture tools hosted by the Center for Clinical and Translational Science and Training. REDCap (Research Electronic Data Capture) is a secure, web-based software platform designed to support data capture for research studies, providing (1) an intuitive interface for validated data capture; (2) audit trails for tracking data manipulation and export procedures; (3) automated export procedures for seamless data downloads to common statistical packages; and (4) procedures for data integration and interoperability with external sources. The data was then exported for further cleaning and analysis to the statistical package of social science (SPSS) version 22. Open-ended questions were initially coded in Microsoft Excel (evaluator 1) and later using SPSS (evaluator 2). To facilitate the process of coding, duplicate cases were first identified. Researchers observed individual participants’ responses for each question to identify text segments of the respondent’s own words. Subsequently, text segments with similar meanings were brought together to generate subthemes. Finally, the themes were generated through further abstraction and grouping of subthemes with the help of a third evaluator (evaluator 3) [30,31].

A grading scale was used to measure participants’ levels of understanding of the definition of health based on whether or not their responses included statements related to the essential domains of human health [32,33]. A “very good understanding” of health should have included statements related to the well-being of three health domains, including physical, mental, and social [34,35]. Those including two domains were considered to have a “good understanding” of health. Those with a “fair understanding” included only a single domain. Those failing to include any of the domains were said to have a “poor understanding” of the definition of health. Ideally, they also would have included the environment as a fourth domain.

Youth participants responding to our survey were grouped into three main groups based on age range (ages 9 to 11 years, 12 to 14 years, and 15 to 18 years) as well as the sample population in its entirety. The normality of data was tested, and comparisons of the groups’ subscales’ scores were made. Demographic data were presented as frequencies (numbers and percentages) or means and standard deviations (SD) where appropriate. Sums and a percentage of the total score for each of the subscales were calculated. Knowledge, self-efficacy, attitude, and behavior percentage scores were presented as mean ± SD when data were normally distributed or median, and interquartile range (IQR) when data were non-normally distributed. We compared the subscales to each other using paired t-tests. We tested associations of respondents’ subscales scores with sex, parent employment status, school type, participation in activities related to environmental health, and history of asthma diagnosis. Student t-test was used for parametric data group comparisons, and the Mann–Whitney (Z) test was used for non-parametric data. Correlations of the studied subscales were assessed using Spearman’s correlation for non-parametric data and Pearson’s correlation for parametric data. With our final quantitative sample size equaling 438, we had 80% power to detect differences as small as 3.74 units between the scores given a standard deviation equaling 27.7 at an alpha equal to 0.05.

## 3. Results

### 3.1. Population Characteristics

A total of 452 participants were included in the qualitative portion of the survey analysis, and 438 youth were included in the quantitative portion of the survey. Their mean age was 13.6 ± 2.6 years. There were more female than male participants, and the proportion of females to males was higher in the 15–18-year-old group compared to either of the other age groups (*p* ≤ 0.020). One of the two participating high schools enrolled only females (Table 1). Most participants considered themselves white, non-Hispanic, and from suburban regions. Nearly two-thirds indicated that both parents worked. A history of asthma was reported in 13.7% of participants. (Table 1). Of the six participating teachers in our study, only one teacher indicated that they directly linked the environment with health in their lessons.

### 3.2. Environmental Health and Health Knowledge

All teachers reported offering lessons about the environment; thus, it was not surprising that most students could verbalize environmental concerns. When asked what worried them most about the environment, air pollution was the most common response (Table 2). Other forms of pollution, such as noise, radiation, soil, and water, were not frequently reported. Within air pollution, tobacco smoking, factories, traffic-related environmental smoke, and global warming were among the primary concerns. Interestingly, those in the 9 to 11-year-old and 12 to 14-year-old age groups more frequently reported smoking/smoke as a concern, while older participants, ages 15 to 18-year-old, tended to report air pollution and global warming as a concern. Other common areas of concern included waste and the impact on the lives of people and animals. However, participants rarely related their environmental concerns to their local environments, including where they live, their own personal activities, or others’ activities that could influence the impact of their environment on their health.

When students were asked how the environment affects people’s health, the themes of air pollution and health/illness status were identified. Participants most cited air pollution as the way the environment would affect their health and make them sick. Of note, participants aged 15 to 18 years mentioned smoking less frequently than the other age groups. Fewer participants pointed out that the environment (e.g., air, water, food, medicine, etc.) contributed to their health/sickness state (health/illness status theme). This is consistent with our participants’ knowledge scores. Importantly, our 438 participants with quantitative responses collectively had a moderate level of knowledge (mean score 66.2 ± 20.6), and these knowledge scores were higher in the two older age groups compared to the younger age group (*p* = 0.001 and *p* < 0.001, respectively).

We then evaluated participants’ open-ended definitions of health. A very good definition of health included physical, mental, and social dimensions, with lower ratings mentioning none, one, or two domains. Only 30 (6.7%) participants had very good definitions. Those with good definitions were primarily (97%) in the 15 to 18-year-old age group (Table 3). Over a quarter of participants had poor definitions or answered “I don’t know” (Table 3), with a significantly higher proportion of these responses being in the 9–11 age group than either of the other age groups (*p* < 0.001). Ideally, a definition of health would also include a mention of the environment. Only nine participants included the environment in their definition of health. Importantly, the inclusion of environment in the definition of health was not related to the quality of health definition (2 very good, 1 good, 5 fair, 1 poor). In addition, there was no significant correlation between the definition of health score rankings and the knowledge score.

### 3.3. Behavior Is Weakly Associated with Environmental Health Knowledge

**Figure 1:**
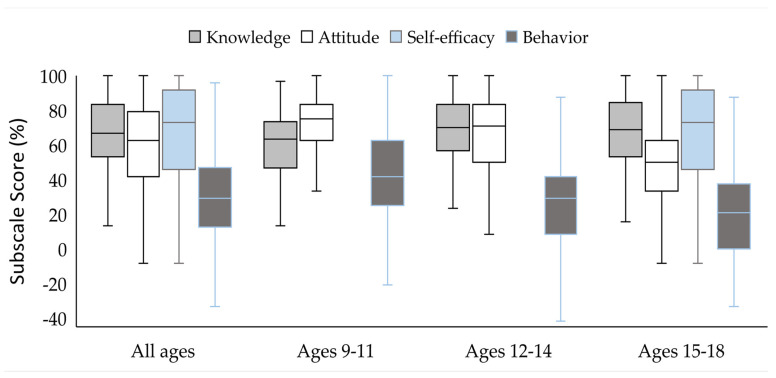
Subscale Scores by Age (Years). Bars represent the median, lower, and upper quartile subscales’ percentage scores within different age groups. Whiskers represent the minimum and maximum subscale score. *n* = 438.

We then sought to gain a better understanding of the participants’ behaviors related to environmental health. When participants were asked how they could help (or protect) the environment, all age groups pointed to cleaning up the environment (almost two-thirds). Other pro-environment behaviors, such as recycling, not smoking, composting, and planting trees, were reported less frequently (less than one-fourth). Participants repeatedly reported that cleaning up and or not littering would help the environment, commonly saying “pick up others’ litter” and “teach others not to litter.” Still, few indicated that they were willing to lead by example. A 9-year-old boy indicated he could “recycle, reuse, reduce, not litter, and pick up litter.” The participants’ quantitative survey behavior scores were similarly low, with a mean score of 29.03 ± 27.7. The behavior scores were lowest in the older age groups (Figure 1; *p* < 0.001).

To gain a complete understanding of why behavior scores were collectively low, we first found that behavior scores were significantly lower than knowledge scores (*p* < 0.001) and were only weakly correlated (r = 0.22, *p* < 0.001), which was consistent across the age groups (Table 4). Next, we considered the relationship between behavior scores and attitude and self-efficacy scores. Similar to knowledge scores, attitude and self-efficacy scores were higher than behavior scores (*p* < 0.001), but the correlations with behavior were moderate (r attitude = 0.54, *p* < 0.001; r self-efficacy = 0.51, *p* < 0.001). Surprisingly, correlations between knowledge scores and attitude and self-efficacy were also moderate (r attitude = 0.38, *p* < 0.001; r self-efficacy = 0.47, *p* < 0.001).

### 3.4. Factors Associated with Behavior Scores

To better understand specific factors associated with behavior scores, we took a closer look at our 15–18-year-old participant group. We found that participation in environment-related classes, activities, and/or clubs was significantly associated with behavior (*p* = 0.006, Table 5). Importantly, involvement in either formal and/or non-formal classes, activities, and/or clubs related to environmental health was associated with significantly higher scores on the other subscales, including knowledge (*p* = 0.003), attitude (*p* = 0.006), and self-efficacy (*p* = 0.02) scores. In contrast, attending private school was only associated with higher attitude scores (*p* = 0.004). The participants with both parents employed had higher behavior scores (*p* = 0.03) as well as higher self-efficacy scores (*p* = 0.045). Participants with histories of asthma had elevated self-efficacy scores (*p* = 0.007).

## 4. Discussion

Environmental challenges lead to serious health problems in the US, especially for children, and action by the lay public is lacking. While there has been substantial work evaluating the relationship between knowledge, attitude, behavior, and self-efficacy, few studies have examined the relationship between these components with respect to environmental health in youth. This study sought to characterize the relationship between environmental health knowledge and behavior in youth. We found variable understanding of environmental health and knowledge exhibiting weak association with behavior in youth. Further, while participants identified environmental concerns, they rarely related these concerns to their local environments. As such, participants often failed to connect environmental concerns to their own health or the health of those within their communities. We also noted that knowledge was only weakly correlated with behavior, but was moderately correlated with attitude, suggesting a complex relationship. Participation in focused environmental activities is associated with improved knowledge and behavior scores. These results suggest that environmental health programming for youth could improve environmental health knowledge and action. This study highlights the need to better connect human health and environmental health for youth. Citizen science programs could help bridge this divide.

It was not unexpected that our study participants’ knowledge scores were generally higher than other scores. Indeed, most youth were able to acknowledge several, albeit general, environmental concerns. A prior study supported our finding that youth identify air pollution as an important environmental concern [34]. Most likely, widespread information about air pollution in the local media has contributed to the salience of air pollution-related issues in our study and others [35]. Another reason for the recognition of air pollution is that Cincinnati has been known to be one of the worst metropolitan regions for year-round particle pollution by the American Lung Association [36]. Still, participants rarely related concerns to their local environments or their own health. Specifically, participants failed to mention environmental concerns about the communities in which they lived. Further, their concerns about air pollution centered around outdoor air quality; indoor air quality was never mentioned, even though it has a well-recognized impact on human health [37]. This disconnect between environmental concerns and a participant’s own health is consistent with a prior study showing difficulties in connecting environmental concerns with specific health outcomes [34]. Our study also observed that participants lacked an understanding of the definition of health, evidenced by a few youths, including the three domains (physical, mental, and social, described by the World Health Organization) [38] of health in their responses. Even fewer youth mentioned the impact of the environment within their definitions.

Our results also demonstrate that knowledge is weakly associated with behavior. While participants could identify pro-environment actions, few reported taking action. Behavior scores were significantly lower than knowledge scores as well. The environmental health literacy framework captures how environmental health knowledge is translated into action [39,40]. Briefly, environmental health literacy has dimensions including awareness and knowledge, skills and self-efficacy, and community change [39]. While knowledge is a core element, it is insufficient to drive change. Previous studies have found that although environmental knowledge is a prerequisite to action, knowledge alone is not enough to promote environmental behaviors [41,42]. Studies evaluating knowledge and behavior found only weak [41] or moderate correlations [43,44]. Our findings were consistent with these studies and suggested that knowledge alone is insufficient to adopt pro-environmental behavior. Rather, correlations between behavior–attitudes–self-efficacy and knowledge–attitudes–self-efficacy were much stronger than correlations between behavior–knowledge. Accordingly, attitudes and self-efficacy may be important factors in making information actionable. Indeed, a meta-analysis demonstrated that interventions that modify attitudes and self-efficacy effectively promote health behavior changes [45]. To positively change attitudes and self-efficacy, environmental health education must become relatable. For example, the COVID-19 pandemic (which occurred after data collection for this study was completed) frequently impaired people’s healthy behaviors [46]. However, the impact of the environment on health has never been more tangible. The negative impact of COVID-related lockdowns and social distancing, as well as the positive impact on outdoor air quality [47] and pediatric asthma exacerbations [48], are observable examples of the relationship between environment and health. However, one must also recognize that the home and school environments must be amenable to pro-environmental health behavior. This is especially relevant for children who live with their parents and may not have control of their environment. Thus, there should be further studies evaluating why individuals do not adopt pro-environmental behaviors.

Our results demonstrate that although knowledge is weakly associated with behavior, targeted environmental health education is associated with improvement in knowledge, behavior, attitudes, and self-efficacy. Indeed, youth who participated in environmental health related classes, clubs, and activities had higher knowledge and behavior scores. This is consistent with a prior study evaluating an environmental health education program, which found improvements in both risk perception and information-seeking behaviors [49]. In our study, only one teacher out of six reported specifically addressing environmental science in the curriculum, while 46% of students reported formal or informal environmental health-related classes, activities, or clubs. Moreover, health is often not included in science courses introducing environmental topics. When we met with the environmental science teacher, this class did not specifically address health. It is important to recognize there may be bias in which youth participants opt to participate in environmental health activities because they may already have more knowledge and interest in environmental action prior to their participation.

Nonetheless, our results are consistent with prior studies indicating that access to environmental education is associated with improvements in knowledge, attitudes, and behavior [50,51]. These improvements may be because learning skills within a context-specific framework ultimately empowers learners to participate in the decision-making process on behalf of the environment and commit to action [52]. Earlier work suggests that young people might benefit from more extracurricular activities if the curriculum has the goal of helping participants understand both the short and long-term effects of the environment on health [15]. Thus, targeted environmental health-focused education programs are likely effective strategies to equip youth with the ability to develop and implement their own contextually appropriate strategies for addressing their personal and community environmental health risks [53,54].

There are several strengths and limitations to our study. First, a unique strength of our study was the inclusion of a diverse age range of youths (9–18 years). While the incorporation of such a broad age range is important for capturing diversity, we captured knowledge, attitudes, and behaviors using a single tool adapted from one originally designed for use with middle schoolers. Due to the developmental changes occurring across these age groups, the use of tools developed specifically for certain age ranges would be optimal. Still, the qualitative component in our study (open-ended questions) complemented our understanding of participants’ levels of knowledge, attitudes, and behaviors across this diverse age group. Additionally, when working with communities exposed to specific environmental health risks, tools will need to be further customized for their unique challenges [55]. More recently, others have created environmental health literacy tools that capture knowledge items deemed to be essential by a large group of environmental health literacy experts [56]. Though we found associations between participation in environment-related classes, clubs, and activities and knowledge, attitudes, behaviors, and self-efficacy, our cross-sectional observational study design does not prove that such participation impacts these outcomes.

While our study was restricted to youth from well-resourced schools, the benefits of environmental education may be greatest in individuals with low socioeconomic status [57]. Non-formal activities, including those involving community and/or citizen science, in particular may benefit students attending underperforming schools. Prior studies have used participatory research approaches to improve health literacy [58,59]. However, it is important to recognize that different communities have different health risks and environmental challenges [40]. As health disparities increase communities’ environmental health risks [50], communities experiencing disparities in health and/or environmental challenges are likely to greatly benefit from “local” environmental health programming [14,16,60]. Thus, relevant social, economic, and political contexts are a must. To this end, co-creating programs with experts from various scientific disciplines, community partners, and citizens from affected communities are critical for successful programming [61]. An important aspect of this co-creation will be the development of harmonized language between the community and academic scientists [62]. Furthermore, engaging children and adolescents in improving public health mutually benefits the growth and development of youth [63] while simultaneously strengthening public health and community development efforts [64,65]. Historically, these young voices have been left out. Yet, because the youth live with families, having intergenerational programs may be of value.

## 5. Conclusions

We concluded that knowledge was weakly associated with behavior and that educational activities positively affected behavior. There is a need for more environmental health education opportunities, including locally focused programming that promotes actionable environmental health knowledge. Citizen science programs could help bridge this divide. However, the complex relationship of knowledge, attitudes, and self-efficacy and how it can influence health/environmental behaviors suggests a need for direct evaluation of the impact of any educational program on these factors.

## Figures and Tables

**Table 1:** Participant Characteristics.

Characteristics		Age Groups (Years), *n* (%)		
	Total (*n* = 452)	9–11 (*n* = 127)	12–14 (*n* = 135)	15–18 (*n* = 190)
Age, years (years)	13.6 ± 2.6	10.1 ± 0.8	13.3 ± 0.6	16.2 ± 0.9
Sex				
Female	268 (59.3)	65 (51.2)	74 (54.8)	129 (67.9)
Male	181 (40.1)	60 (47.2)	60 (44.4)	61 (32.1)
Hispanic/Latino Ethnicity				
Yes	22 (4.9)	8 (6.3)	5 (3.7)	9 (4.7)
No	424 (93.8)	118 (92.9)	128 (94.8)	178 (93.7)
Race				
White	411 (90.9)	119 (93.7)	123 (91.1)	169 (88.9)
Black/African American	3 (0.7)	0	2 (1.5)	1 (0.5)
Asian/Pacific Islander	5 (1.1)	1 (0.8)	1 (0.7)	3 (1.6)
Native American	5 (1.1)	2 (1.6)	2 (1.5)	1 (0.5)
Other	24 (5.3)	4 (3.2)	7 (5.2)	13 (6.8)
Residence				
Rural	28 (6.2)	5 (3.9)	5 (3.7)	18 (9.5)
Suburban	395 (87.4)	114 (89.8)	124 (91.9)	157 (82.6)
Urban	26 (5.8)	7 (5.5)	4 (2.9)	15 (7.9)
Parents’ employment status				
Both parents unemployed	11 (2.4)	3 (2.4)	2 (1.5)	6 (3.2)
Both parents employed	297 (65.7)	73 (57.5)	94 (69.6)	130 (68.4)
One parent employed	111 (24.6)	25 (19.7)	35 (25.9)	51 (26.8)
Do not know	32 (7.1)	26 (20.5)	3 (2.2)	3 (1.6)
Asthma history				
Yes	62 (13.7)	14 (11.1)	19 (14.1)	29 (15.3)
No	386 (85.4)	112 (88.2)	114 (84.4)	160 (84.2)

Note: Information on sex, residence, race, and Hispanic/Latino status is missing in 3, 3, 4, 6 cases, respectively. Parent employment status and asthma history are missing in 1 and 4 cases, respectively. *n*, frequency.

**Table 2:** Qualitative Themes and Subthemes (*n* = 452).

Questions	Themes	Subthemes and Youth Quotes
What worries you about the environment?	Air pollution	Smoking: Something that worries me about the environment is when people like smokers start polluting the air. Also, some trains pollute the air when they go down the train tracks (10- year old female).
		Environmental smoke: Air pollution worries me about the environment because there will be too much smoke in the earth (9-year old male).
		Global warming: I hear about global warming often, and it scares me that so many people don’t take care of the environment (16-year old female).
	Waste	Littering: When people litter in parks or even just on the street (9-year old female)
		Overfull landfills: I worry about global warming and things that sit in the landfill forever when they could be biodegradable somewhere else, e.g., compost pile, back yard (12-year old female).
	Wild and human life	Health impact: That people will get sick, and more animals will die because of pollution (13-year old male).
		Plant and animal loss: All the polluted air and oil in the water that are harming animals and plants (18-year old female).
How or in what ways does the environment affect health?	Air pollution	Smoking: If someone around you is smoking and you breathe in that air a lot, you could get sick (9-year old female).
		Environmental smoke: Pollution, such as smoke, makes it difficult to breathe (15-year old female).
	Health and illness state	Human needs from the environment: The environment keeps me healthy and strong; If there aren’t many trees, we wouldn’t have oxygen. The environment affects my health by giving me air, water, and food; I have allergies to some of the plants and things. It gives me allergies, colds, and sometimes fevers (9-year old female).
What can you do to help (or protect) the environment?	Do not pollute	Clean up/not littering I can pick up litter and help others become more aware of the environment (13-year old male).
		Pro-environmental behaviors: I can pick up litter and help others become more aware of the environment (13-year old male).
	Save resources	Save wildlife: I can recycle, try not to waste electricity, and try not to waste water, plant trees or other plants, and many other things (14-year old male).
		Save energy/not waste: I can use less water and just basically reduce my carbon footprint by recycling and using clean fuel systems like wind power or hydroelectricity (18-year old female).

**Table 3:** Rating of Definitions of Health in the cohort and by age group.

	Age Group (Years)			
Rating (*n* (%) within Age Group)	All (*n* = 448)	9–11 (*n* = 125)	12–14 (*n* = 134)	15–18 (*n* = 189)
Don’t Know	13 (2.9)	10 (8.0)	1 (0.7)	2 (1.1)
Poor	103 (23.0)	54 (43.2)	26 (19.4)	23 (12.2)
Fair	264 (58.9)	56 (44.8)	93 (69.4)	115 (60.8)
Good	38 (8.5)	5 (4.0)	13 (9.7)	20 (10.6)
Very Good	30 (6.7)	0 (0)	1 (0.7)	29 (15.3)

**Table 4:** Correlations of Participants’ Subscales by Age Group.

Age Groups (y)	Behavior & Knowledge	Behavior & Attitude	Behavior & Self-efficacy	Knowledge & Attitude	Knowledge & Self-Efficacy	Self-Efficacy & Attitude
All (*n* = 442)	r = 0.22 **	r = 0.54	r = 0.51 **	r = 0.38 **	r = 0.47 **	r = 0.50 **
9–11 (*n* = 127) 12–14 (*n* = 123)	r = 0.39 ** r = 0.29 **	r = 0.59 ** r = 0.41 **	------ ------	r = 0.51 ** r = 0.55 **	------ ------	------ ------
15–18 (*n* = 188)	r = 0.21 **	r = 0.49 **	r = 0.51 **	r = 0.45 **	r = 0.47 **	r = 0.50 **

Note: r, correlation coefficient; y, year; Spearman and Pearson correlation tests were used. ** Statistically significant at *p* ˂ 0.001 level. Responses from a single child in the 15–18 y age group is missing for knowledge subscale.

**Table 5:** Factors associated with Environmental Health Sub-scores in Participants Ages 15–18 Years.

	Behaviors	Knowledge	Attitudes	Self-Efficacy
Participation in environment-related classes, activities, and/or clubs				
Yes (*n* = 86)	26.2 ± 25.3	73.9 ± 20.9	54.5 ± 20.8	75.0 (35.4)
No (*n* = 102)	16.3 ± 23.4	65.1 ± 19.1	44.6 ± 23.3	66.6 (45.8)
	*t* = 2.8, ***p* = 0.006**	*t* = 3.03, ***p* = 0.003**	*t* = 3.03, ***p* = 0.003**	Z = 2.3, ***p* = 0.02**
School Type *				
Private (*n* = 56)	24.8 ± 25.4	73.4 ± 17.5	56.8 ± 18.8	80.5 (41.7)
Public (*n* = 73)	19.0 ± 23.1	67.2 ± 21.2	46.4 ± 20.7	70.8 (43.8)
	*t* = 1.3, *p* = 0.2	*t* = 1.7, *p* = 0.08	*t* = 2.9, ***p* = 0.004**	Z = 1.4, *p* = 0.2
Parent employment				
2 parents working (*n* = 129)	20.8 (37.5)	71.3 ±19.4	48.8 ± 22.2	75.0 (41.7)
One parent working (*n* = 50)	16.7 (34.4)	65.2 ± 21.6	51.9 ± 23.2	56.3 (51.0)
	Z = 2.2, ***p* = 0.03**	*t* = 1.8, *p* = 0.07	*t* = 0.8, *p* = 0.4	Z = 2, ***p* = 0.045**
Asthma status				
Asthmatic (*n* = 29)	25% ± 25.9	72.3% ± 20.2	49.2% ± 24.3	91.7 (35.4)
No asthma (*n* = 157)	19.8% ± 24.1	68.7% ± 20.5	49.1% ± 22.5	66.6 (41.7)
	*t* = 1.1, *p* = 0.3	*t* = 0.9, *p* = 0.4	*t* = 0.007, *p* = 0.9	Z = 2.7, ***p* = 0.007**

* Female participants. Student (*t*) tests and Mann–Whitney (Z) tests were used. *p*-values in bold were considered statistically significant (*p* < 0.05).

## Data Availability

The datasets generated and analyzed during the study are available from the corresponding authors on reasonable request.
